# Identifying Candidate Polyphenols Beneficial for Oxidative Liver Injury through Multiscale Network Analysis

**DOI:** 10.3390/cimb46040193

**Published:** 2024-04-02

**Authors:** Sang Yun Han, Ji-Hwan Kim, Gi-Sang Bae, Won-Yung Lee

**Affiliations:** 1 The Office of Korean Medicine Education, College of Korean Medicine, Daejeon University, Daejeon 34530, Republic of Korea; 2 Department of Sasang Constitutional Medicine, Division of Clinical Medicine, School of Korean Medicine, Pusan National University, Busan 46241, Republic of Korea; 3 Department of Pharmacology, College of Korean Medicine, Wonkwang University, Iksan 54538, Republic of Korea; 4 Research Center of Traditional Korean Medicine, Wonkwang University, Iksan 54538, Republic of Korea; 5 Department of Pathology, College of Korean Medicine, Wonkwang University, Iksan 54538, Republic of Korea; 6 Department of Pathology, College of Korean Medicine, Woosuk University, Jeon-Ju 54987, Republic of Korea

**Keywords:** oxidative liver injury, polyphenols, multiscale network

## Abstract

Oxidative stress, a driver of liver pathology, remains a challenge in clinical management, necessitating innovative approaches. In this research, we delved into the therapeutic potential of polyphenols for oxidative liver injury using a multiscale network analysis framework. From the Phenol-Explorer database, we curated a list of polyphenols along with their corresponding PubChem IDs. Verified target information was then collated from multiple databases. We subsequently measured the propagative effects of these compounds and prioritized a ranking based on their correlation scores for oxidative liver injury. This result underwent evaluation to discern its effectiveness in differentiating between known and unknown polyphenols, demonstrating superior performance over chance level in distinguishing these compounds. We found that lariciresinol and isopimpinellin yielded high correlation scores in relation to oxidative liver injury without reported evidence. By analyzing the impact on a multiscale network, we found that lariciresinol and isopimpinellin were predicted to offer beneficial effects on the disease by directly acting on targets such as CASP3, NR1I2, and CYP3A4 or by modulating biological functions related to the apoptotic process and oxidative stress. This study not only corroborates the efficacy of identified polyphenols in liver health but also opens avenues for future investigations into their mechanistic actions.

## 1. Introduction

Oxidative liver injury is a significant concern in liver disease management, involving the complex role of reactive oxygen species (ROS) in both physiological and pathological conditions. ROS can adversely affect critical cellular components, including proteins, lipids, and DNA, leading to a range of cellular responses from growth stimulation to death, activating defense mechanisms against microorganisms, and modulating gene expression in signal-transduction pathways [1,2]. The liver, one of the body’s largest organs, is central to regulating energy metabolism, detoxifying substances, storing nutrients, and managing the immune system [3,4]. However, oxidative stress can impair liver function, resulting in a variety of liver diseases marked by symptoms such as fatigue, anorexia, indigestion, jaundice, and bleeding tendencies. These conditions may arise from autoimmune, drug-induced, alcoholic, infectious, and congenital metabolic disorders, all initiated by oxidative stress and subsequent inflammatory damage [5,6,7]. Given the pivotal role of oxidative damage in liver diseases and their prevalence, there is a clear gap in effective treatment options. This gap underscores the necessity of identifying potential new approaches to address the molecular mechanisms of oxidative liver injury, offering a pathway to developing more effective treatments for these conditions. This study introduces a novel approach by employing multiscale network analysis to identify potential polyphenols that could mitigate oxidative liver injury, thereby addressing a critical gap in current therapeutic strategies.

Network pharmacology, emerging from the realm of systems biology, has established itself as a crucial tool in the discovery and development of new therapeutic strategies. This approach has illuminated the path to identifying rational drug targets and repurposing existing medications by mapping the complex interplay within biological networks [8,9]. It challenges the traditional single-target paradigm, offering a multifaceted view of disease treatment through the lens of interconnected biological networks [10,11]. This is particularly relevant for natural products and herbal medicines, known for their multi-compound, multi-target properties, allowing for a deeper understanding of their mechanisms. For example, network pharmacology has shed light on the action of cordycepin against breast cancer, as well as the effects of the guizhi-fuling capsule in treating primary dysmenorrhea, through comprehensive network analysis and experimental validation [12,13]. Furthermore, Ruiz et al. proposed that considering propagation effects on a multiscale network can effectively identify the therapeutic effects of a drug [14]. Such advancements underscore network pharmacology’s potential in enhancing our understanding of drug–disease interactions and in pinpointing active compounds within herbal medicine, paving the way for innovative treatment options.

Building on the potential of network pharmacology, we explored candidate polyphenols beneficial for oxidative liver disease through multiscale network analysis. To this end, we meticulously selected polyphenols that met our criteria, exploring their chemical distribution and the frequency of their targets. Subsequently, we prioritized those polyphenols believed to be effective against oxidative liver injury based on their propagative effects within the multiscale network. To test the reliability of our predictions, we conducted recapitulating tests to ascertain whether our results could distinguish between known and unknown polyphenols with efficacy against liver disease. Additionally, we delved into the top-prioritized polyphenols, seeking out reported evidence of their effectiveness. Finally, we analyzed the mechanisms of high-scoring, yet unreported, polyphenols within the multiscale network. We believe that our approach not only illuminates the potential of multiscale network analysis in the discovery of novel therapeutic compounds but also sets the stage for future research into the polyphenol mechanisms.

## 2. Materials and Methods

### 2.1. Compound-Target Network Construction

Information on polyphenols and their target interactions was systematically compiled and interconnected, with polyphenolic data being sourced from the Phenol-Explorer database (version 3.6) [15,16]. This database catalogues polyphenols, providing comprehensive food composition data and profiling these compounds in biofluids following interventions with polyphenol-rich diets. Specifically, attention was directed towards 501 polyphenols for which composition data were available, ensuring that a thorough investigation into their dietary sources and potential health benefits was conducted. For the purpose of our analysis, selection criteria were strictly adhered to, including only those polyphenols for which mapping to PubChem IDs was feasible.

Protein targets were derived from comprehensive datasets encompassing compound-target interactions that had been experimentally validated, as well as predictions from the Swiss Target Prediction platform. The data on experimentally confirmed targets were sourced from several reputable databases, including DrugBank, the Therapeutic Target Database (TTD), the Search Tool for Interactions of Chemicals (STITCH), and a compilation by Huang et al. [17,18,19,20]. These resources collectively offer extensive details on established and potential targets, diseases they are associated with, pathways involved, and drugs aimed at these specific targets. STITCH compiles target data for over 430,000 chemicals, drawing from various sources. Huang et al.’s compilation focuses on both direct and indirect interactions between compounds and proteins, specifically for natural products, drawing from a broad range of databases. Swiss Target Prediction serves as a web-based tool for predicting potential targets of small bioactive molecules. To ensure accurate target identification, symbols such as gene symbols or UniProt IDs were aligned with Entrez Gene IDs through the use of SYNGO [21].

### 2.2. Disease-Associated Protein

Proteins implicated in liver damage and oxidative stress were identified through the use of the STRING database query functionality within the Cytoscape StringApp plugin [22]. This tool leverages text-mining technologies to generate a network of proteins that are relevant to specific search queries. Its efficacy in isolating proteins linked to particular diseases through targeted mechanisms has been demonstrated, such as in research aiming to highlight proteins involved in Parkinson’s disease by concentrating on neuroinflammation aspects [23]. For this task, the search terms ‘liver injury’ AND ‘oxidative stress’ were employed. Proteins or genes that exceeded a certain co-occurrence score threshold were earmarked as being pertinent to liver injury in the context of oxidative stress. The benchmark for this selection was set at a score of 1, which represents a stricter criterion than that used in some prior investigations [23]. These proteins were then incorporated into Cytoscape 3.7 as a STRING network, laying the groundwork for further network analysis.

### 2.3. Multiscale Interactome Construction

The comprehensive interactome utilized in our research was adapted from the work of Ruiz et al. [14]. This network was constructed by weaving together three categories of connections: interactions between proteins, between proteins and biological functions, and among biological functions themselves. For the protein–protein interactions component, the assembly included 387,626 physical connections spanning 17,660 proteins, sourced from key repositories, such as the Biological General Repository for Interaction Datasets, the Database of Interacting Proteins, and the Human Reference Protein Interactome Mapping Project. Regarding the protein–biological function links, a total of 34,777 associations were mapped, linking 7993 proteins to 6387 biological functions, as delineated in the human edition of the Gene Ontology database. Lastly, for the interrelations between biological functions, a structured hierarchy was developed, featuring 22,545 associations across 9798 distinct biological functions.

### 2.4. Diffusion Profile Calculation and Analysis

Active components relevant to the disease were pinpointed by computing and assessing their diffusion profiles, which encapsulate the impact of drugs or diseases on proteins and biological functions. The diffusion profile, represented as $r\;\in {\mathbb{R}}^{\left|V\right|}$, was derived using a matrix-based method incorporating power iteration in the following manner:

$$r^{\left(k+1\right)}=\left(1-\alpha \right)s+\alpha \left(r^{\left(k\right)}M+s\underset{j\in J}{\sum }r_{j}^{\left(k\right)}\right)$$

where $r^{\left(k\right)}$ symbolizes the diffusion profile at the kth state; α is the probability of the walker continuing; $s\;\in {\mathbb{R}}^{\left|V\right|}$ is a restart vector that indicates the likelihood of the walker jumping to each node after restarting, and M represents a directed multiscale network-derived biased transition matrix along with a set of scalar weights highlighting the probability of visiting other nodes.

The iterative process was maintained until the power iteration stabilized, satisfying the following condition:

$$||\;r^{\left(k+1\right)}-r^{\left(k\right)}\;|{|}_{1}>\varepsilon$$

with ε set as the tolerance parameter at $1\times 10^{-6}$, aligning with previous studies. 

To compute the correlation between the drug and disease diffusion profiles, the following formula was employed:

$$\frac{\left(r^{\left(c\right)}-{\bar{r}}^{\left(c\right)}\right)\;\cdot \;\left(r^{\left(d\right)}-{\bar{r}}^{\left(d\right)}\right)}{||(r^{\left(c\right)}-{\bar{r}}^{\left(c\right)})|{|}_{2}\;||\left(r^{\left(d\right)}-{\bar{r}}^{\left(d\right)}\right)|{|}_{2}}$$

where *r*^(*c*)^ and *r*^(*d*)^ denote the diffusion profiles for the drug and disease, respectively.

Key mechanisms of the compound–disease pair were discerned by examining their diffusion profiles, selecting the top k-proteins or biological functions based on their impact from the drug or disease. A network of these selected entities was then constructed to illuminate their significance. Any compound targets not linked to disease-associated proteins or biological functions were omitted. The entity with the highest rank in the diffusion profile was considered most critical for treatment due to its significant influence. The value of k was determined as 20 to adequately reflect a substantial portion of the visitation frequency within the diffusion profile.

### 2.5. Assessment of Multiscale Network-Based Prediction Results on Polyphenol

Polyphenols with therapeutic effects on liver disease were systematically identified from the Comparative Toxicogenomics Database [24], specifically under the category of liver disease (MeSH ID: D008107). Eleven polyphenols were selected based on their documented therapeutic actions. These compounds were then analyzed within a multiscale network to assess their correlation scores, which served to distinguish between known and hitherto unreported polyphenols regarding their therapeutic efficacy in liver disease.

The evaluation of the classification model was conducted objectively using two standard metrics: AUROC and AUPR. The AUROC provides a measure of the model’s ability to discriminate between classes across all thresholds, with a higher area indicating better performance. Conversely, the AUPR reflects the precision and recall of the model, particularly informative when the classes are imbalanced. The areas under these curves were calculated to quantify the model’s accuracy in differentiating the polyphenols’ therapeutic potential, thus validating the model’s predictive strength in a multiscale network context.

## 3. Results

### 3.1. Selection and Analysis of Polyphenols and Their Targets

**Figure 1:**
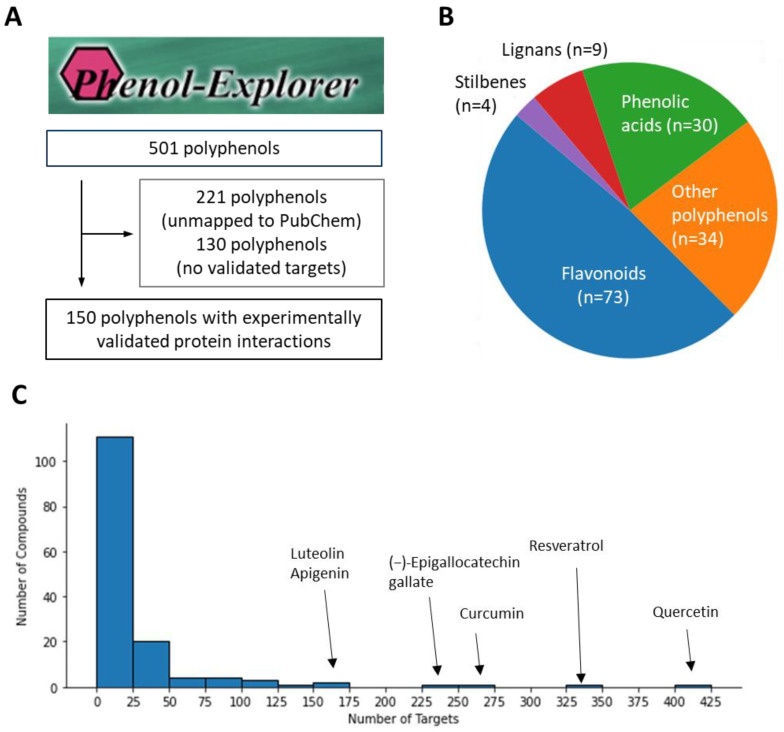
Distribution and target profile of selected polyphenols. (**A**) Polyphenol selection process from Phenol-Explorer database. (**B**) Chemical class distribution of selected polyphenols. (**C**) Target frequency profile of polyphenols.

We first implemented a selection process to identify polyphenols with validated targets for addressing oxidative liver injury. Utilizing the extensive Phenol-Explorer database as our primary resource, we extracted an initial list of 501 polyphenols [15,16]. Our criteria for selection included the presence of a PubChem Chemical ID (CID) and sufficient reported target interactions. After this initial screening, we excluded 221 polyphenols lacking a PubChem CID and a further 130 compounds with insufficient target data, ultimately yielding a focused group of 150 viable polyphenols for subsequent analysis (Figure 1A). We further visualized the chemical class distribution and found that they showed a diverse range of compounds, with flavonoids constituting the majority at 48.7%. Phenolic acids, lignans, and stilbenes followed, reflecting the varied potential for therapeutic intervention within these classes (Figure 1B). In exploring the target distribution among the included polyphenols, we observed that approximately three-quarters (74.5%) interacted with fewer than 25 targets. This suggests a high degree of specificity within most compounds’ target profiles. On the other hands, certain polyphenols demonstrated an extensive range of interactions, with quercetin leading at 424 targets. Following were resveratrol, curcumin, (−)-epigallocatechin gallate, luteolin, and apigenin with 326, 251, 226, 164, and 151 targets, respectively (Figure 1C). This thorough screening provided us with a reliable set of compounds and their target information, crucial for our subsequent analysis.

### 3.2. Network Construction and Enrichment Analysis

**Figure 2:**
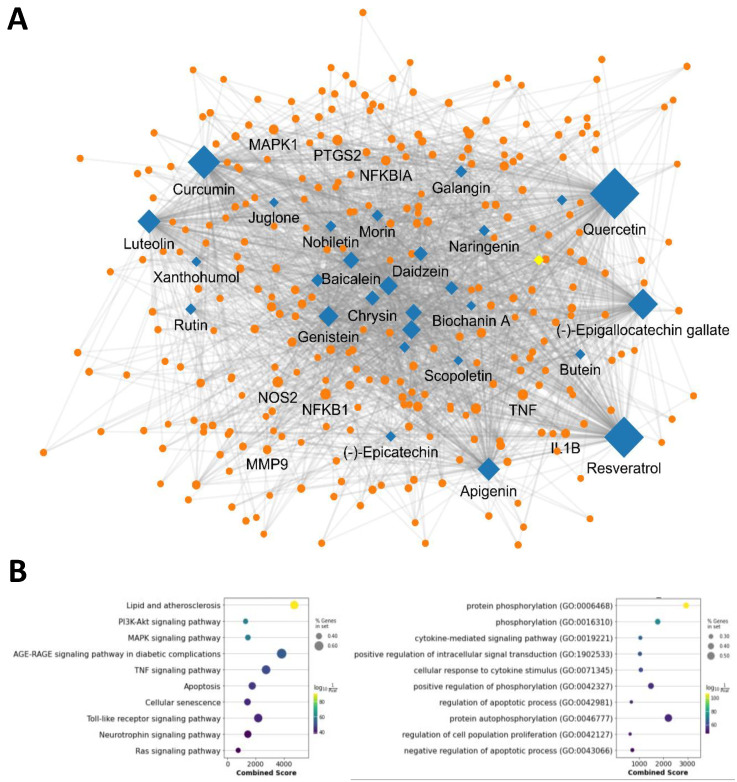
Polyphenol-target network and associated signaling pathway and biological function. (**A**) Polyphenol-target interaction network. Diamonds and circles represent polyphenols and targets, respectively. (**B**) Pathway and biological function enrichment of polyphenol targets. Bubble chart depicts pathways and biological functions linked to polyphenol targets. The x-axis shows the association significance, bubble size reflects the gene count involved, and color indicates the statistical significance of each pathway or function.

We then visualized the network by mapping the interactions between polyphenols and their targets that exceeded the mean frequency of occurrence. The network comprised 34 polyphenolic compounds and their 279 associated targets, resulting in 2065 linkages (Figure 2A). This intricate network highlights the multi-target properties of polyphenols, suggesting their potential to exert beneficial effects through a wide array of biological interactions. To further investigate these interactions, we conducted an enrichment analysis to identify the signaling pathways and biological functions associated with the polyphenols. Our analysis revealed significant associations with several key signaling pathways, notably lipid and atherosclerosis, PI3K-Akt, MAPK, AGE-RAGE, and TNF signaling pathways (Figure 2B, left panel). Additionally, we observed a strong association with essential biological functions such as protein phosphorylation, cytokine-mediated signaling, and cellular response to cytokine stimulus, as well as the regulation of apoptotic processes (Figure 2B, right panel). These findings underscore the comprehensive role of polyphenols in modulating molecular pathways and support the rationale for their potential use in therapeutic strategies for oxidative liver injury.

### 3.3. Identification of Polyphenols for Oxidative Liver Injury Using Multiscale Network Analysis

**Figure 3:**
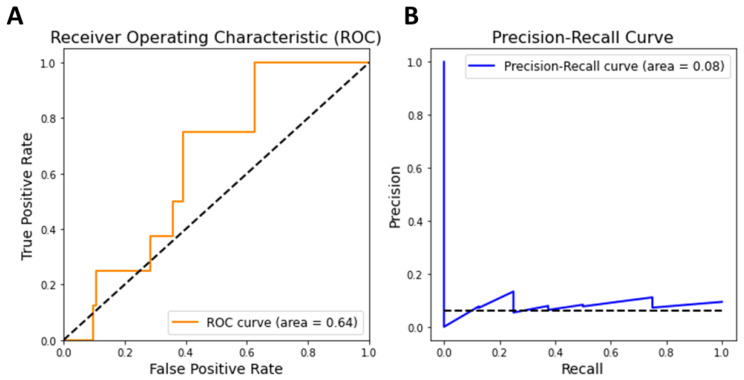
Performance curves of multiscale network-based prediction for polyphenol targets. (**A**) Receiver Operating Characteristic (ROC) Curve. The ROC Curve (orange line) with an area under the curve (AUC) of 0.64 indicates the model’s ability to distinguish between known and unknown polyphenol targets for liver disease. (**B**) Precision-Recall Curve. The dashed black line in both curves represents the chance level performance.

To explore potential polyphenols, we calculated diffusion profiles for all polyphenols in relation to oxidative liver injury and ranked the polyphenols based on their correlation scores. Polyphenols with high correlation scores are likely to be of higher priority for utilization in oxidative liver injury. To assess the reliability of these prediction scores, we first examined whether multiscale network-based scores could effectively distinguish between 11 polyphenols known to affect oxidative liver injury, as listed in the Comparative Toxicogenomics Database, and other polyphenols. The discriminative performance was measured using two metrics: the Area Under the Receiver Operating Characteristic (AUROC) and the Area Under the Precision-Recall Curve (AUPR). Note that we focused on polyphenols with sufficient targets (three or more) to ensure a level of reliability in our predictions. The results showed that our multiscale network-based scores yielded an AUROC of 0.64 and an AUPR of 0.08 (Figure 3). These scores suggest that our predictive model can reliably identify potential polyphenol candidates for the treatment of oxidative liver injury, as demonstrated by its ability to discern established effective compounds from a broader set.

We collated the top ten polyphenols, prioritized based on their correlation scores, and calculated the overlap of these compounds with proteins associated with oxidative liver injury, along with a search for reported evidence of efficacy (Table 1). The results demonstrated that all polyphenols had *p*-values below the threshold of 1 × 10^−5^, substantiating the capacity of multiscale network-based prediction to identify polyphenols with targets significantly connected to oxidative liver disease. Furthermore, we found that 8 out of these top 10 polyphenols had already been reported to exert beneficial effects on the disease. This not only reinforces the predictive precision of our multiscale network approach but also suggests its potential utility as a reliable metric for discovering polyphenols that may confer therapeutic benefits in the context of oxidative liver injury.

### 3.4. Multiscale Network Level Mechanisms for Polyphenol Candidates

**Figure 4:**
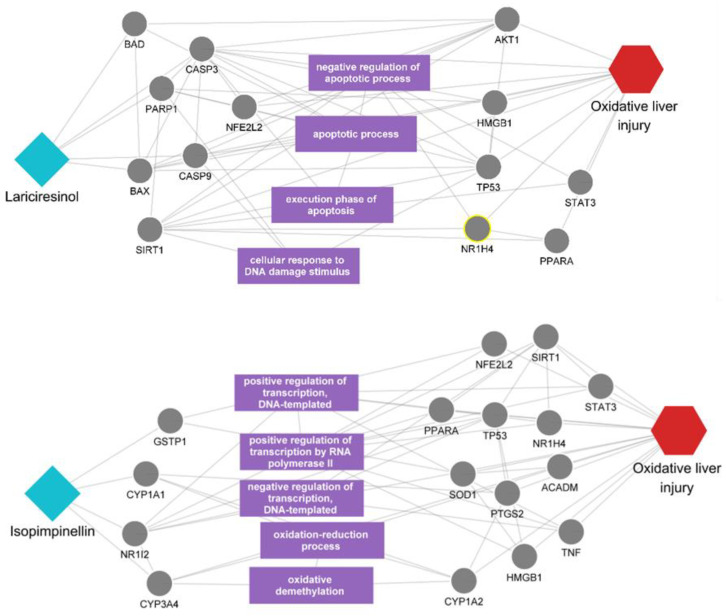
Multiscale network mechanisms of lariciresinol and isopimpinellin in oxidative liver injury. The figure presents the proposed mechanisms of action for lariciresinol and isopimpinellin in the context of oxidative liver injury within a multiscale network. Diamond shapes represent the polyphenols lariciresinol and isopimpinellin. Circles denote the target proteins and biological processes associated with each polyphenol. Hexagons indicate the disease of interest. The purple rectangle highlights distinct biological processes.

The outcome of our prioritization process suggests that the remaining unreported polyphenols could represent novel candidates with the potential to exert beneficial effects on oxidative liver injury. To validate this proposition, we delved into the multiscale network mechanisms of these polyphenols, aiming to uncover the underlying interactions that may contribute to their therapeutic action (Figure 4). In our predictive analysis, we initially focused on lariciresinol, which emerged as a highly scored yet unreported candidate. It was observed that lariciresinol can directly affect the disease-related proteins such as CASP3, CASP9, and BAX. Also, it was found to interact with a variety of proteins, such as BAD, CASP3, PARP1, and BAX, which are engaged in a network of interactions with other proteins linked to the disease. Furthermore, these proteins are involved in the regulation of several biological processes related to apoptosis, including the negative regulation of the apoptotic process and the execution phase of apoptosis. Following lariciresinol, our analysis visualized the impact of isopimpinellin, another polyphenol with predictive relevance to the disease. Isopimpinellin demonstrated the potential to directly affect disease-related targets such as NR1I2 and CYP3A4. Additionally, it appeared to influence biological functions related to oxidative stress, such as the oxidation-reduction process and oxidative demethylation, suggesting a capacity to modify the function of proteins involved in the disease process through these mechanisms.

## 4. Discussion

In our study, we have successfully navigated the complex landscape of polyphenols to identify promising candidates for oxidative liver injury using a multiscale network-based analysis approach. We started by rigorously selecting polyphenols with validated targets, analyzing their chemical class distribution and target frequency. The subsequent enrichment analysis provided further insight into the biological functions and signaling pathways associated with these compounds. Our methods successfully differentiated known effective polyphenols from lesser-known ones, suggesting the reliability of our predictive model. We then probed deeper into the multiscale-level mechanisms of unreported polyphenols, uncovering promising new candidates for mitigating oxidative liver injury. This discussion will aim to contextualize these findings within the broader landscape of liver disease research, emphasizing the novel insights our study provides and the implications for future therapeutic development.

Our research capitalized on the strengths of multiscale network analysis, a method that elucidates the extensive effects of drugs and diseases on the human interactome. Building upon previous work that explored candidate flavonoids for non-alcoholic fatty liver disease through a network proximity-based strategy [25], our study advances this research by incorporating the effects of propagation on a multiscale network. Following simulation of propagation effects on a multiscale network, we leveraged a biased random walk algorithm to calculate the similarity between these interactions [14]. This algorithm, pivotal across fields for its ability to assess the influence of nodes within complex networks, is particularly beneficial in biology for predicting therapeutic effects of small molecules and natural products [26]. The predictive accuracy of our analysis was fine-tuned by adjusting transition probabilities, favoring movements toward biological functions over proteins. Indeed, most of the top-ranked polyphenols have been previously reported to offer benefits for liver health, which validates the effectiveness of our multiscale network-based approach. Our results signify not only the robustness of our model in identifying polyphenol candidates for treating oxidative liver injury but also the utility of the multiscale network approach in discerning intricate biological interactions and their therapeutic implications. 

We have successfully navigated the complex landscape of polyphenols to identify promising candidates for oxidative liver injury using a multiscale network-based analysis approach. Our analysis highlighted lariciresinol as a top-scoring compound among unreported polyphenols. Lariciresinol, characterized by its presence in sesame seeds, Brassica vegetables, and the bark and wood of white fir, is a precursor to enterolignans, which are speculated to exhibit beneficial medicinal properties due to the action of gut microflora. Previous research suggested lariciresinol’s significant antioxidant properties, specifically through the activation of the Nrf2-mediated heme oxygenase-1 (HO-1) expression, which plays a pivotal role in cellular defense against oxidative stress [27]. The findings from our multiscale network-based analysis are consistent with previous research that has identified the antioxidant capabilities of lariciresinol. Notably, our predictive analysis revealed lariciresinol’s potential to directly influence proteins like CASP3, CASP9, and BAX, which are implicated in the cellular defense mechanisms against oxidative stress. These interactions and the compound’s involvement in modulating processes related to apoptosis align with its suggested role in activating Nrf2-mediated HO-1 expression, a crucial pathway for mitigating oxidative damage within liver tissues. As we explore the therapeutic potential of identified polyphenols like lariciresinol and isopimpinellin, it is pertinent to consider recent advancements in flavonoid formulations, including water-soluble forms and nanoparticles. These innovations, demonstrating enhanced neuroprotective properties and bioavailability, may further amplify the beneficial effects of polyphenols in oxidative liver injury management [28,29].

Our analysis identified isopimpinellin as another noteworthy polyphenol with untapped potential for addressing oxidative liver injury. Isopimpinellin is naturally synthesized by various plant species, particularly those in the carrot family, Apiaceae, including celery, garden angelica, parsnip, and in the rind and pulp of limes. This compound has been explored for its anticarcinogenic properties, notably its potential inhibitory effects on skin tumor initiators like 7,12-dimethylbenz(a)anthracene and its possible linkage to the inhibition of breast cancers [30]. The observed activities of isopimpinellin within our analysis align with its known biological impacts. We found that isopimpinellin could directly engage with targets such as NR1I2 and CYP3A4, which are central to the body’s response to oxidative liver injury. These interactions, coupled with its influence on processes pivotal to oxidative stress management like oxidation–reduction and oxidative demethylation, correlate with previously noted anti-carcinogenic properties, underscoring its potential to alter protein function and contribute to therapeutic outcomes against liver diseases.

## 5. Conclusions

Our study leverages the comprehensive approach of multiscale network analysis to illuminate the complex interplay of polyphenols and their targets in the context of oxidative liver injury, providing a novel perspective on potential therapeutic strategies. By identifying and analyzing both known and previously unreported polyphenols, we demonstrate the utility of this approach in discovering compounds with significant therapeutic potential. Notably, compounds like lariciresinol and isopimpinellin emerged as promising candidates, underscoring the importance of multiscale network analysis in advancing our understanding of natural products and their mechanisms of action. This work underscores the importance of exploring natural products through advanced analytical techniques, offering a straightforward yet effective approach to identifying new treatments for liver disease.

## Figures and Tables

**Table 1:** Prioritized polyphenols related to oxidative liver disease.

Compound Name	PubChem CID	Correlation Score	Overlap (*p*-Value #)	Reported Evidence (PMID)
Jaceosidin	5379096	0.159	5/5 (0)	18449499
Bisdemethoxycurcumin	5315472	0.116	3/4 (1.3 × 10^−8^)	25594342
Lariciresinol	332427	0.102	3/5 (6.44 × 10^−8^)	.
Isovitexin	162350	0.101	3/5 (6.44 × 10^−8^)	29604422
Myricitrin	5281673	0.100	9/17 (2.49 × 10^−16^)	25656916
Astilbin	119258	0.098	4/7 (2.78 × 10^−9^)	33780953
Oleuropein	5281544	0.097	4/7 (2.78 × 10^−9^)	21145829
Isoorientin	114776	0.091	5/11 (6.11 × 10^−10^)	35185572
Isopimpinellin	68079	0.090	2/4 (4.91× 10^−6^)	.
Punicalagin	16129869	0.089	5/11 (6.11 × 10^−10^)	11351354

‘#’ symbol next to the *p*-value indicates that the value was derived using the hypergeometric test, a statistical method used to determine the significance of the overlap between two sets of data.

## Data Availability

The datasets supporting the conclusions of this article are included within the article.
